# Effectiveness of antenatal intravenous immunoglobulin treatment in recurrent fetal and neonatal alloimmune thrombocytopenia

**DOI:** 10.1002/uog.70105

**Published:** 2025-10-15

**Authors:** K. Zloto, S. Cohen, L. Batsry, A. Schwartz, T. Weissbach, H. Avnet, B. Weisz, Y. Yinon

**Affiliations:** ^1^ Department of Obstetrics and Gynecology Sheba Medical Center Tel Hashomer Israel; ^2^ School of Medicine, Faculty of Medical and Health Sciences Tel Aviv University Tel Aviv Israel

**Keywords:** fetal and neonatal alloimmune thrombocytopenia, FNAIT, severe thrombocytopenia

## Abstract

**Objective:**

To evaluate the effectiveness of antenatal intravenous immunoglobulin (IVIG) treatment for recurrent fetal and neonatal alloimmune thrombocytopenia (FNAIT) and to determine factors associated with a good response to treatment.

**Methods:**

This was a retrospective cohort study of pregnant women diagnosed with FNAIT and managed at a single tertiary referral center between 1992 and 2024. Affected pregnancies were treated non‐invasively with weekly IVIG (1 g/kg maternal body weight) from 18–24 weeks until delivery, without platelet count monitoring. All cases were monitored with serial ultrasound for intracranial hemorrhage (ICH). Fetal blood sampling was offered at 37 weeks' gestation to determine the mode of delivery; vaginal delivery was allowed when the fetal platelet count was ≥ 50 000/*μ*L. A good response to treatment was defined by the absence of ICH and a platelet count ≥ 50 000/*μ*L at birth. Neonatal and maternal outcomes were assessed, and clinical characteristics were compared between pregnancies with a good response to treatment *vs* non‐responders.

**Result:**

During the study period, 91 women (225 pregnancies) were diagnosed with FNAIT. Of these, 121 (53.8%) pregnancies were treated with IVIG: 48 patients had one IVIG‐treated pregnancy, 17 had two, six had three, four had four and one patient had five consecutive IVIG‐treated pregnancies. Only two women were treated during their first affected pregnancy based on family history; the remaining 89 women were diagnosed after the delivery of an affected neonate. IVIG treatment was declined in 15/134 (11.2%) subsequent pregnancies. Among the IVIG‐treated pregnancies, 100/121 (82.6%) exhibited a good response to IVIG, with a median platelet count at birth of 172 000/*μ*L, while 21/121 (17.4%) did not respond to treatment, with a median platelet count at birth of 27 000/*μ*L. None of the treated pregnancies was complicated by ICH. Human platelet antigen (HPA)‐1a alloimmunization was significantly more common in the group of non‐responders (95.2% *vs* 60.0%, *P* < 0.01), while HPA‐5 alloimmunization was significantly more common among those who responded to treatment (29.0% *vs* 4.8%, *P* = 0.02). A comparison of consecutive IVIG‐treated pregnancies (totaling 115 pregnancies) revealed no significant differences between the first, second and third pregnancy with respect to platelet count at birth. However, among 11 women with at least three IVIG‐treated pregnancies, four exhibited variable responses to treatment, with some pregnancies resulting in severe thrombocytopenia (platelet count < 50 000/*μ*L) despite a good response in the preceding pregnancy. Five (5.5%) patients experienced ICH, all during their first untreated pregnancy. In subsequent pregnancies, no case of ICH was reported in either treated or untreated pregnancies.

**Conclusion:**

A good response to IVIG in one FNAIT‐affected pregnancy does not guarantee successful treatment in subsequent pregnancies. The reason why IVIG may be effective in one pregnancy but ineffective in a later pregnancy for the same patient remains unclear. © 2025 The Author(s). *Ultrasound in Obstetrics & Gynecology* published by John Wiley & Sons Ltd on behalf of International Society of Ultrasound in Obstetrics and Gynecology.

## INTRODUCTION

Fetal and neonatal alloimmune thrombocytopenia (FNAIT), defined as a platelet count of < 50 000/μL^1^, is the leading cause of severe thrombocytopenia in newborns and is a major contributor to intracranial hemorrhage (ICH)^2^. The incidence of FNAIT is approximately 1 in 1000 births^3^. FNAIT occurs when the maternal immune system produces antibodies against paternally derived platelet‐specific antigens that are present on fetal but not maternal platelets^2^, ^4^. The most common antigen associated with FNAIT in Caucasian populations is human platelet antigen (HPA)‐1a (75–80%), followed by HPA‐5b (10–15%)^5^. While HPA‐1a incompatibility is the most frequent cause, more than 30 other platelet antigen mismatches can cause FNAIT, although these are less common and typically result in milder disease^6^. Maternal exposure to incompatible fetal platelet antigens triggers an immune response, leading to the production of maternal antibodies that cross the placenta and cause fetal thrombocytopenia^7^, ^8^. Symptoms range from incidental findings of asymptomatic thrombocytopenia, to mild bleeding, such as petechiae or purpura, to more serious complications like ICH, which can occur antenatally or within the first 24–48 h after birth^9^, ^10^.

Currently, there are no routine screening programs for FNAIT, and diagnosis usually occurs after birth when thrombocytopenia is identified or symptoms appear^11^. Diagnosis is confirmed by identifying maternal–fetal HPA incompatibility and corresponding maternal antibodies^8^, ^12^, ^13^.

The recurrence rate of FNAIT is approximately 90%, but the disease does not necessarily worsen in subsequent pregnancies^12^, ^14^. In women with a previous child affected by ICH, the reported recurrence rate for antenatal ICH in a future pregnancy without treatment varies widely, from 60% to nearly 100%^15^, ^16^, ^17^, ^18^. Treatment involves weekly maternal infusions with intravenous immunoglobulin (IVIG), sometimes combined with oral steroids^19^. This treatment, while effective, is not supported by placebo‐controlled trials, and management is based on clinical presentation in a previous pregnancy, with tailoring of treatment according to the anticipated severity of FNAIT^2^, ^20^, ^21^. Moreover, there is some evidence to suggest that IVIG treatment may not be necessary in all cases^19^.

There is insufficient information within the existing literature on the effectiveness of IVIG treatment in pregnancies with recurrent FNAIT, with respect to fetal platelet count response. This study aimed to investigate the effectiveness of antenatal IVIG treatment in recurrent FNAIT and to determine whether a favorable response to IVIG in one FNAIT pregnancy predicts a similar response in subsequent affected pregnancies. Additionally, we aimed to identify the factors associated with a good response to treatment.

## METHODS

This was a retrospective cohort study of all pregnant women diagnosed with FNAIT and managed at Sheba Medical Center, a single tertiary referral center in Tel Hashomer, Israel, between 1992 and 2024. A review of electronic medical records was conducted to identify all eligible individuals during the study period. The study was approved by the ethics committee of Sheba Medical Center, which waived the requirement for informed consent owing to its retrospective nature (1415‐24‐SMC‐D).

Data were obtained from a comprehensive computerized perinatal database. Maternal characteristics collected included age, body mass index and use of fertility treatment. Obstetric characteristics, such as hypertensive disorders of pregnancy, gestational diabetes mellitus, placental abruption and preterm prelabor rupture of membranes, were also assessed. Delivery records were examined for the use of predelivery cordocentesis and mode of delivery. Neonatal records were reviewed for sex, birth weight, birth‐weight percentile (calculated using Israeli birth‐weight curves^22^), platelet count at birth and the presence of petechiae, ecchymoses and/or ICH.

**Figure 1:**
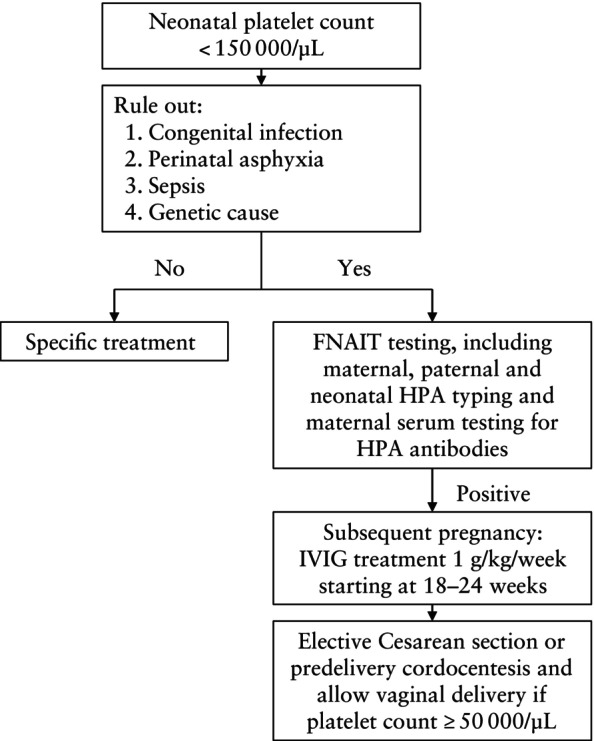
Flowchart summarizing diagnosis and management of fetal and neonatal alloimmune thrombocytopenia (FNAIT). Each neonate in the first affected pregnancy underwent thorough assessment, including complete physical examination, blood tests, cultures and, if a genetic cause was suspected, genetic testing. HPA, human platelet antigen; IVIG, intravenous immunoglobulin.

In most cases, the diagnosis of FNAIT was suspected following the delivery of a first affected child with either symptomatic or asymptomatic neonatal thrombocytopenia (defined as a platelet count of < 150 000/μL). After ruling out other potential causes of neonatal thrombocytopenia (Figure 1), postnatal confirmation of the diagnosis was achieved through parental and neonatal HPA typing, which revealed incompatibility between the mother and the father/newborn, accompanied by maternal antibody testing, which confirmed the presence of corresponding antibodies. Laboratory analysis included platelet crossmatching via the indirect platelet immunofluorescence test, monoclonal antibody‐specific immobilization of platelet antigens, solid‐phase enzyme‐linked immunosorbent assay, DNA isolation and HPA genotyping using a TaqMan assay.

In subsequent pregnancies, if the father was homozygous for the implicated antigen, the fetus was assumed to be affected. In cases in which the father was heterozygous, second‐trimester diagnostic amniocentesis was performed for fetal genotyping.

In two cases, the diagnosis was suspected during the first pregnancy owing to a family history of FNAIT involving the patient's sister. These cases received IVIG treatment during the first pregnancy.

Affected pregnancies were managed non‐invasively with weekly administration of IVIG at a dose of 1 g/kg of maternal body weight, without platelet count monitoring, from 18–24 weeks' gestation until delivery. In cases of treatment failure with the standard IVIG dose in a previous pregnancy, treatment was initiated at an earlier gestational age and with a higher dose of IVIG (1.5–2 g/week), with or without corticosteroids at a daily dose of 0.5 mg/kg. Owing to the higher risk associated with cordocentesis in the presence of fetal thrombocytopenia, we adopted a non‐invasive approach of empiric IVIG treatment without performing cordocentesis before term. All cases were monitored with monthly neurosonography starting at 28 weeks to detect signs of fetal ICH. At 37 weeks, all patients were offered either elective Cesarean section or fetal blood sampling (by cordocentesis) to determine the mode of delivery. Cordocentesis was performed in a double setup within the operating room. Vaginal delivery was permitted if the fetal platelet count was ≥ 50 000/μL. Operative vaginal delivery was not permitted. Following cordocentesis, fetal cardiotocography was conducted for 1 h. Postnatal cranial ultrasound was performed in all newborns. A good response to treatment with IVIG was defined by two criteria: the absence of ICH and a neonatal platelet count at birth of ≥ 50 000/μL.

Continuous variables were analyzed using the non‐parametric Mann–Whitney U‐test, while categorical variables were compared using the chi‐square test. ANOVA was conducted to compare continuous variables between the first, second and third consecutive IVIG‐treated pregnancies. Subgroup analysis was performed for individuals with IVIG‐treated pregnancies to compare clinical characteristics between those who responded well to IVIG *vs* non‐responders. All statistical tests were two‐tailed, and *P* < 0.05 was considered statistically significant. Statistical analysis was performed using SPSS version 25 (IBM Corp., Armonk, NY, USA).

## RESULTS

During the study period, 91 individuals (225 pregnancies) were diagnosed with FNAIT. Among these, 121 (53.8%) pregnancies received IVIG treatment. Specifically, 48 women had one IVIG‐treated pregnancy, 17 women had two consecutive IVIG‐treated pregnancies, six women had three, four women had four and one woman had five consecutive IVIG‐treated pregnancies. Only two women were treated during their first affected pregnancy based on family history; the remaining 89 women were diagnosed after the delivery of an affected neonate. IVIG treatment was declined in 15/134 (11.2%) subsequent pregnancies; none of these cases had a history of ICH.

The clinical characteristics of the first affected pregnancies are shown in Table 1. The median neonatal platelet count at birth was 20 500/μL. Platelet count at birth was < 50 000/μL in 56 (61.5%) patients and < 30 000/μL in 51 (56.0%) patients. Thirty (33.0%) neonates presented with petechiae or ecchymoses, and five (5.5%) neonates experienced ICH.

**Table 1:** Clinical characteristics of 91 first pregnancies complicated by fetal and neonatal alloimmune thrombocytopenia

Characteristic	Value
Alloimmunization	
HPA‐1a	58 (63.7)
HPA‐2	14 (15.4)
HPA‐3	20 (22.0)
HPA‐5	31 (34.1)
HPA‐15	26 (28.6)
Platelet count at birth (/μL)	20 500 (13 000–60 250)
Platelet count at birth < 50 000/μL	56 (61.5)
Platelet count at birth < 30 000/μL	51 (56.0)
Neonatal petechiae/ecchymoses	30 (33.0)
Intracranial hemorrhage	5 (5.5)

Data are given as *n* (%) or median (interquartile range). Two women were treated with intravenous immunoglobulin based on positive family history; the remaining 89 women were diagnosed after delivery of the affected neonate. HPA, human platelet antigen.

The clinical characteristics of the IVIG‐treated pregnancies are presented in Table 2. Four (3.3%) were twin pregnancies; all twin pairs were born with concordant platelet counts (126 000/μL and 147 000/μL; 72 000/μL and 81 000/μL; 150 000/μL and 160 000/μL; 303 000/μL and 291 000/μL). The median gestational age at initiation of IVIG treatment was 21 weeks, and that at delivery was 37.6 weeks. Predelivery cordocentesis was performed in 43 (35.5%) cases, of which 35 (81.4%) resulted in normal vaginal delivery and eight (18.6%) in Cesarean section owing to severe fetal thrombocytopenia (platelet count was < 50 000/μL in seven cases and in one case it was 58 000/μL). At birth, 21 (17.4%) neonates had a platelet count of < 50 000/μL and 12 (9.9%) had a platelet count of < 30 000/μL. Six (5.0%) neonates had petechiae or ecchymoses, and none experienced ICH.

**Table 2:** Clinical characteristics of 121 pregnancies complicated by fetal and neonatal alloimmune thrombocytopenia and treated with intravenous immunoglobulin (IVIG)

Characteristic	Value
Maternal age (years)	33 (29–36)
Prepregnancy BMI (kg/m^2^)	23 (20–26)
Twin pregnancy	4 (3.3)
Conception by IVF	3 (2.5)
Hypertensive disorder of pregnancy	6 (5.0)
Gestational diabetes mellitus	15 (12.4)
Placental abruption	1 (0.8)
PPROM	6 (5.0)
GA at starting IVIG treatment (weeks)	21 (19–25)
Steroid treatment	17 (14.0)
GA at delivery (weeks)	37.6 (36.6–38.5)
Predelivery cordocentesis	43 (35.5)
NVD after cordocentesis	35/43 (81.4)
CS for NRFHR after cordocentesis	0/43 (0)
CS for thrombocytopenia after cordocentesis	8/43 (18.6)
CS	76 (62.8)
NVD	45 (37.2)
Platelet count at birth (/μL)	147 000 (72 000–240 000)
Platelet count at birth < 50 000/μL	21 (17.4)
Platelet count at birth < 30 000/μL	12 (9.9)
Neonatal petechiae/ecchymoses	6 (5.0)
Intracranial hemorrhage	0 (0)
Platelet transfusion	1 (0.8)
Female neonatal sex	55 (45.5)
Birth weight (g)	2907 (2620–3180)
Birth‐weight percentile	50.5 (33.7–64.2)

Data are given as median (interquartile range), *n* (%) or *n/N* (%). BMI, body mass index; CS, Cesarean section; GA, gestational age; IVF, *in‐vitro* fertilization; NRFHR, non‐reassuring fetal heart rate; NVD, normal vaginal delivery; PPROM, preterm prelabor rupture of membranes.

Among the 15 pregnancies for which IVIG treatment was declined, nine (60.0%) had a platelet count of < 50 000/μL at birth, and four (26.7%) neonates presented with petechiae.

Among the 121 IVIG‐treated pregnancies, 100 (82.6%) showed a good response to IVIG, with a neonatal platelet count at birth of ≥ 50 000/μL (median, 172 000/μL), while the remaining 21 (17.4%) did not respond to IVIG treatment, with a platelet count at birth of < 50 000/μL (median, 27 000/μL). A comparison between the two groups is presented in Table 3. A significantly higher proportion of non‐responders had HPA‐1a as the offending antigen (95.2% *vs* 60.0%, *P* < 0.01), whereas a significantly higher proportion of pregnancies that responded well to treatment had HPA‐5 as the offending antigen (29.0% *vs* 4.8%, *P* = 0.02). The neonatal platelet count in the first affected pregnancy was significantly lower in non‐responders (10 500/μL *vs* 18 000/μL, *P* < 0.01); consequently, in this group, IVIG treatment was initiated earlier (median, 19.5 weeks *vs* 22 weeks, *P* < 0.01) and had a longer duration (median, 17 weeks *vs* 16 weeks, *P* = 0.01). There was no significant difference between responders and non‐responders in the rate of predelivery cordocentesis (35.0% *vs* 38.1%, *P* = 0.94). As expected, the rate of normal vaginal delivery was significantly higher in pregnancies that responded well to treatment (43.0% *vs* 9.5%, *P* < 0.01); severe thrombocytopenia precluded normal vaginal delivery in most non‐responders.

**Table 3:** Clinical characteristics of 121 pregnancies complicated by fetal and neonatal alloimmune thrombocytopenia and treated with intravenous immunoglobulin (IVIG), according to whether they responded to treatment

Characteristic	Responded to IVIG (*n* = 100)	Did not respond to IVIG (*n* = 21)	*P*
Maternal age (years)	33 (30–36)	32 (29–33.5)	0.11
Gravidity	4 (3–6)	4 (3–6)	0.85
Parity	2 (1–4)	2 (2–3.5)	0.93
Platelet count at birth in first affected pregnancy (/μL)	18 000 (12 750–50 000)	10 500 (7250–17 500)	< 0.01
Neonatal petechiae/ecchymoses in first affected pregnancy	19 (19.0)	11 (52.4)	0.01
ICH in first affected pregnancy	4 (4.0)	1 (4.8)	1.00
Alloimmunization			
HPA‐1a	60 (60.0)	20 (95.2)	< 0.01
HPA‐2	0 (0)	0 (0)	NA
HPA‐3	15 (15.0)	2 (9.5)	0.51
HPA‐5	29 (29.0)	1 (4.8)	0.02
HPA‐15	13 (13.0)	0 (0)	0.08
GA at start of IVIG treatment (weeks)	22 (20–26)	19.5 (18–21.5)	< 0.01
Duration of IVIG administration (weeks)	16 (11–18)	17 (16–18)	0.01
Amniocentesis	41 (41.0)	2 (9.5)	< 0.01
Steroid treatment	12 (12.0)	5 (23.8)	0.13
GA at delivery (weeks)	38 (37–38.5)	37 (36–38)	0.14
Predelivery cordocentesis	35 (35.0)	8 (38.1)	0.94
CS for thrombocytopenia after cordocentesis	1/35 (2.9)	7/8 (87.5)	< 0.01
NVD after cordocentesis	34/35 (97.1)	1/8 (12.5)	< 0.01
NVD	43 (43.0)	2 (9.5)	< 0.01
Platelet count at birth (/μL)	172 000 (106 500–256 250)	27 000 (12 000–40 000)	< 0.01
Platelet transfusion	1 (1.0)	0 (0)	0.65
Female neonatal sex	47 (47.0)	8 (38.1)	0.46
Birth weight (g)	2960 (2647.5–3245)	2753 (2514–3162.5)	0.18
Birth‐weight percentile	51 (32–66)	44.5 (29–58)	0.16

Data are given as median (interquartile range), *n* (%) or *n/N* (%). CS, Cesarean section; GA, gestational age; HPA, human platelet antigen; ICH, intracranial hemorrhage; NA, not applicable; NVD, normal vaginal delivery.

**Figure 2:**
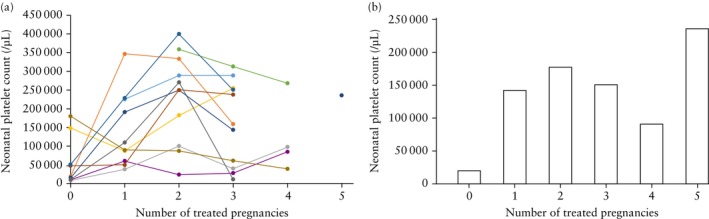
(a) Neonatal platelet count at birth in consecutive pregnancies treated with antenatal intravenous immunoglobulin (IVIG) for recurrent fetal and neonatal alloimmune thrombocytopenia (FNAIT), among 11 women with at least three consecutive treated pregnancies. 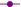, Patient 1; 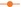, Patient 2; 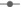, Patient 3; 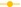, Patient 4; 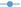, Patient 5; 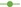, Patient 6; 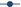, Patient 7; 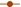, Patient 8; 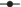, Patient 9; 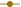, Patient 10; 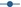, Patient 11. (b) Median neonatal platelet count at birth in consecutive pregnancies treated with antenatal IVIG for recurrent FNAIT, in the same 11 patients. Pregnancy number ‘0’ indicates first pregnancy affected by FNAIT for which IVIG treatment was not administered.

Table 4 compares the characteristics of consecutive IVIG‐treated pregnancies, comprising a total of 115 pregnancies. No significant differences were found between the first, second and third consecutive pregnancies in terms of IVIG treatment characteristics and outcomes. However, a more variable response was observed among the 11 women with at least three IVIG‐treated pregnancies (Table S1, Figure 2a). Seven patients showed consistently good responses to treatment. In contrast, patients 1, 3, 9 and 10 exhibited variable responses, with some pregnancies resulting in severe thrombocytopenia (platelet count < 50 000/μL) despite a good response in the preceding pregnancy, and others resulting in a good outcome despite a poor response in the previous pregnancy. Median platelet count at birth in each successive IVIG‐treated pregnancy is shown in Figure 2b.

**Table 4:** Characteristics of first three consecutive pregnancies complicated by fetal and neonatal alloimmune thrombocytopenia and treated with antenatal intravenous immunoglobulin (IVIG)

Characteristic	First pregnancy (*n* = 76)	Second pregnancy (*n* = 28)	Third pregnancy (*n* = 11)	*P*
Maternal age (years)	32 (29–35)	33 (29–35)	33 (32–36)	0.25
GA at start of IVIG treatment (weeks)	21 (19–25)	20 (19–24)	22 (18–25)	0.50
Duration of IVIG administration (weeks)	16 (11–17.5)	17.5 (14–19)	15 (12.5–16.5)	0.07
Amniocentesis	25 (32.9)	12 (42.9)	4 (36.4)	0.75
Steroid treatment	7 (9.2)	6 (21.4)	2 (18.2)	0.25
GA at delivery (weeks)	37.5 (36.6–38.4)	37.6 (36.5–39.0)	38.2 (36.7–39.4)	0.22
Predelivery cordocentesis	30 (39.5)	10 (35.7)	1 (9.1)	0.16
CS for thrombocytopenia after cordocentesis	6/30 (20.0)	2/10 (20.0)	0/1 (0)	0.88
NVD after cordocentesis	24/30 (80.0)	8/10 (80.0)	1/1 (100)	0.88
CS	49 (64.5)	18 (64.3)	6 (54.5)	0.96
Platelet count at birth (/μL)	142 500 (70 250–218 250)	177 000 (70 500–254 000)	151 000 (37 000–263 500)	0.83
Platelet count at birth < 50 000/μL	13 (17.1)	5 (17.9)	3 (27.3)	0.58
Platelet count at birth < 30 000/μL	8 (10.5)	2 (7.1)	2 (18.2)	0.51
Neonatal petechiae/ecchymoses	5 (6.6)	0 (0)	1 (9.1)	0.32
Intracranial hemorrhage	0 (0)	0 (0)	0 (0)	NA
Platelet transfusion	1 (1.3)	0 (0)	0 (0)	0.80
Female neonatal sex	36 (47.4)	12 (42.9)	5 (45.5)	0.95
Birth weight (g)	2885 (2605–3145)	3010 (2720–3242.5)	3167.5 (2814–3669.5)	0.06
Birth‐weight percentile	48 (31–64)	55 (37.5–67.5)	61 (48–84.5)	0.25

Data are given as median (interquartile range), *n* (%) or *n/N* (%). CS, Cesarean section; GA, gestational age; NA, not applicable; NVD, normal vaginal delivery.

Patient 1 had an adequate response to IVIG in her first treated pregnancy (platelet count at birth, 60 000/μL), but in her second and third IVIG‐treated pregnancies, the platelet count at birth was < 30 000/μL. In her fourth IVIG‐treated pregnancy, the response was adequate, with a platelet count of 85 000/μL at birth. Owing to a lack of response in her second treated pregnancy, prednisone was added at 30 weeks to the routine regimen of IVIG in her third treated pregnancy. In her third treated pregnancy, Cesarean section was performed at 33 weeks because of fetal anemia secondary to fetomaternal hemorrhage. In her fourth treated pregnancy, owing to a refractory response to treatment in previous pregnancies, IVIG at a dose of 1 g/kg was initiated at 20 weeks and the dose was increased to 1.5 g/kg between 28 and 36 weeks alongside steroids.

Patient 3 had a thrombocytopenic infant with a platelet count of 38 000/μL at birth in her first treated pregnancy, an adequate response in her second treated pregnancy (platelet count at birth, 100 000/μL) and another thrombocytopenic infant in her third treated pregnancy (platelet count at birth, 40 000/μL). Owing to a lack of response in the preceding pregnancy, 1 g/kg/week IVIG was initiated at 18 weeks and 0.5 mg/kg/day prednisone was administered from 29 weeks in her fourth treated pregnancy. Prednisone was stopped at 33 weeks because of fetal cataract, and IVIG was increased to 2 g/kg/week from 35 weeks until delivery at 38 weeks, resulting in an adequate platelet count of 97 000/μL at birth.

Patient 9 exhibited HPA‐3 incompatibility in all pregnancies. She responded well in her first two treated pregnancies but, in her third, the infant was born with a platelet count of 11 000/μL and petechiae despite 17 weeks of IVIG treatment.

Patient 10 delivered a thrombocytopenic infant after three successful IVIG‐treated pregnancies. In this patient, response to treatment declined in each successive pregnancy. Platelet count at birth was 90 000/μL, 87 000/μL, 61 000/μL and 39 000/μL in the first, second, third and fourth treated pregnancies, respectively.

Table 5 presents a comparison between patients with HPA‐1a incompatibility and those with other HPA types. The median gestational age at initiation of IVIG treatment was significantly lower in patients with HPA‐1a‐mediated FNAIT (20 weeks *vs* 25 weeks, *P* < 0.01), and the median duration of IVIG administration was longer in the HPA‐1a group (17 weeks *vs* 11.5 weeks, *P* < 0.01). The rate of normal vaginal delivery was significantly lower among patients with HPA‐1a incompatibility (28.8% *vs* 53.7%, *P* < 0.01). Additionally, the median platelet count at birth was significantly lower in the HPA‐1a group (100 000/μL *vs* 186 000/μL, *P* < 0.01). No case of ICH was observed in either group.

**Table 5:** Clinical characteristics of 121 pregnancies complicated by fetal and neonatal alloimmune thrombocytopenia and treated with antenatal intravenous immunoglobulin (IVIG), according to causative human platelet antigen (HPA)

Characteristic	HPA‐1a (*n* = 80)	All other HPA (*n* = 41)	*P*
Maternal age (years)	32 (29–36)	33 (29–36)	0.66
GA at start of IVIG treatment (weeks)	20 (18–22)	25 (22–28)	< 0.01
Duration of IVIG administration (weeks)	17 (15–18)	11.5 (7.5–16)	< 0.01
Steroid treatment	10 (12.5)	7 (17.1)	0.47
GA at delivery (weeks)	37.4 (36.4–38.2)	38.1 (37.1–39.2)	0.01
Predelivery cordocentesis	25 (31.3)	18 (43.9)	0.12
Normal vaginal delivery	23 (28.8)	22 (53.7)	< 0.01
Platelet count at birth (/μL)	100 000 (45 000–216 000)	186 000 (146 500–272 000)	< 0.01
Neonatal petechiae/ecchymoses	4 (5.0)	2 (4.9)	1.00
Intracranial hemorrhage	0 (0)	0 (0)	NA
Female neonatal sex	36 (45.0)	19 (46.3)	0.84
Birth weight (g)	2865 (2595–3150)	3015 (2834.5–3561)	< 0.01
Birth‐weight percentile	49 (33–60)	53 (35–79)	0.17

Data are given as median (interquartile range) or *n* (%). GA, gestational age; NA, not applicable.

Adverse effects associated with IVIG use were generally mild but, in 14 (11.6%) cases, discontinuation of treatment or early delivery was required. These included allergic rash (*n* = 4), IVIG‐induced elevated liver function test (*n* = 8), transient ischemic attack (*n* = 1) and aseptic meningitis (*n* = 1).

## DISCUSSION

This study demonstrates that a successful response to IVIG treatment in one pregnancy with FNAIT does not guarantee the same outcome in subsequent pregnancies. A comparison between the first, second and third consecutive IVIG‐treated pregnancies revealed no significant differences in clinical characteristics, including platelet count at birth, suggesting that the response is generally similar across consecutive pregnancies and that no specific factors can predict the response from one pregnancy to another. However, in the subset of 11 pregnancies with at least three IVIG‐treated pregnancies, a variable response was observed.

The primary goal of antenatal management of FNAIT is to prevent severe thrombocytopenia and, consequently, ICH, which can lead to *in‐utero* or postnatal death or long‐term disability^19^, ^23^. Over the years, the antenatal treatment of FNAIT has evolved, thanks in large part to case series outlining outcomes following various management approaches, including serial intrauterine platelet transfusions, weekly IVIG and immunosuppression with corticosteroids^4^, ^16^. Nowadays, IVIG is considered the standard treatment, with effectiveness ranging between 56% and 92% in preventing severe thrombocytopenia at birth^2^, ^16^, ^24^. However, the efficacy of antenatal IVIG treatment has yet to be evaluated in a randomized placebo‐controlled clinical trial, and the ethical justification for conducting such a trial is still debatable^2^, ^25^.

In Norway, the management strategy for FNAIT has differed from that of most other countries, with IVIG treatment reserved only for women who have previously had a child with ICH^26^. In a retrospective study comparing IVIG‐treated and untreated patients, Ernstsen *et al*.^16^ concluded that omitting antenatal IVIG treatment in HPA‐1a‐alloimmunized low‐risk pregnancies (i.e. no prior ICH in siblings) may not increase the risk of ICH in the newborn. Similarly, a review by Kjeldsen‐Kragh *et al*.^20^ concluded that administering IVIG to all pregnant women with low‐risk FNAIT leads to substantial overtreatment with minimal clinical benefit.

Our study aimed to examine whether a previous response to IVIG can predict treatment outcome in subsequent pregnancies. The reason why IVIG is effective in one pregnancy but may fail in another in the same patient remains unclear. One hypothesis is that tolerance to IVIG may develop after successful treatment in previous pregnancies. While we did not identify specific significant factors that could influence or predict the treatment response, we describe cases in which there was a positive response in one pregnancy, but no response in a subsequent pregnancy. However, after adding steroids, changing the IVIG brand or increasing the IVIG dosage, these patients usually showed a better response in the following pregnancy.

We evaluated an additional hypothesis of an association between response to IVIG and fetal sex. However, no significant difference in fetal sex distribution was observed between pregnancies that responded to treatment and non‐responders (Table 3). Similarly, analyzing outcomes across successive pregnancies showed a comparable proportion of female fetuses (~45%) in all groups (Table 4).

Several studies have explored the combination of steroids with IVIG in the treatment of FNAIT. However, there is no consensus on whether adding steroids to the treatment regimen improves pregnancy outcomes^24^. Berkowitz *et al*.^26^ conducted a randomized, multicenter study involving 40 pregnant women whose previous children had peripartum ICH or whose current fetus had an initial platelet count of < 20 000/μL. These women were assigned randomly to receive either IVIG plus prednisone or IVIG alone. The combination of IVIG and prednisone was clearly more effective compared with IVIG alone in achieving a satisfactory fetal platelet response.

Our management of FNAIT included the option of predelivery cordocentesis at 37 weeks to determine the mode of delivery (if the platelet count was ≥ 50 000/μL, vaginal delivery was allowed). This approach is innovative and resulted in vaginal delivery in 81% of patients who underwent cordocentesis, with favorable outcomes and no cases of ICH. There are various strategies to determine mode of delivery. The Norwegian management approach recommends elective Cesarean section 1–2 weeks prior to term for pregnancies with high anti‐HPA‐1a antibody levels (≥ 3 IU/mL) and vaginal delivery when antibody levels are low (< 3 IU/mL)^25^. Kamphuis and Oepkes^2^ proposed vaginal delivery for multiparous women with a non‐traumatic previous delivery and no history of a child with ICH.

Our study population closely mirrors FNAIT cohorts described in the literature, with a 17.4% rate of non‐response to treatment. In most studies reporting on IVIG treatment for FNAIT, approximately 20% of fetuses do not seem to respond, with platelet counts remaining below 50 000/μL^2^. Additionally, we can assume that non‐responders had more severe disease, as indicated by a significantly higher rate of HPA‐1a alloimmunization, which is a known poor prognostic factor for FNAIT^19^. These patients also required a longer duration of treatment and initiation of IVIG therapy at an earlier gestational age. Previous work has shown that HPA‐1a antibody level can predict the severity of disease in FNAIT pregnancies^27^. However, the measurement of HPA antibody level was not part of our management protocol and, to date, there are no data on the value of HPA antibody level in the prediction of response to IVIG treatment.

Our study has several limitations. Maternal anti‐HPA antibody levels were not measured, therefore, their role in predicting response to treatment could not be assessed. Furthermore, the study's retrospective design introduces the potential for biases inherent to this type of research. The strength of this study lies in its single‐center design, ensuring meticulous documentation of pregnancy follow‐up and outcome. Furthermore, the current literature lacks data on the effectiveness of IVIG treatment in recurrent FNAIT‐affected pregnancies, making this study the first to focus on this unique population. An additional strength is the low incidence of ICH in the cohort, indicating that the study population was predominantly of a standard risk level, better representing the overall FNAIT population.

In conclusion, our study shows that a good response to IVIG in one FNAIT‐affected pregnancy does not guarantee the same response in subsequent pregnancies. The reasons for this variability remain unclear and may be related to the development of tolerance to IVIG after successful treatment in previous pregnancies. Further research is needed to explore this phenomenon.

## Supporting information


**Table S1** Characteristics of 11 patients with at least three consecutive pregnancies complicated by fetal and neonatal alloimmune thrombocytopenia and treated with antenatal intravenous immunoglobulin (IVIG).

## Data Availability

The data that support the findings of this study are available from the corresponding author upon reasonable request.
