# Supplementary material for: Effectiveness of antenatal intravenous immunoglobulin treatment in recurrent fetal and neonatal alloimmune thrombocytopenia
Source: Ultrasound Obstet Gynecol. 2025 Oct 15;66(6):773–80. doi: 10.1002/uog.70105 (PMC12671933; doi:10.1002/uog.70105)
Supplement: Supplementary file 1 — Table S1 Characteristics of 11 patients with at least three consecutive pregnancies complicated by fetal and neonatal alloimmune thrombocytopenia and treated with antenatal intravenous immunoglobulin (IVIG). [file UOG-66-773-s001.docx]

**Table S1** Characteristics of 11 patients with at least three consecutive pregnancies complicated by fetal and neonatal alloimmune thrombocytopenia and treated with antenatal intravenous immunoglobulin (IVIG)

| **Platelet count at birth- (/μL)** | | **Mode of delivery** | **GA at delivery (weeks)** | **Steroid treatment** | **IVIG brand type** | **IVIG dose**  **(g/kg)** | **Duration of tratment (weeks)** | **GA upon starting treatment (weeks)** | **Alloimmu-nization** | **Pregnancies** |
| --- | --- | --- | --- | --- | --- | --- | --- | --- | --- | --- |
|  | |  |  |  |  |  |  |  |  | Patient 1 |
| 9000 | |  |  |  |  |  |  |  | HPA-1a | 1^st^ affected not treated  pregnancy |
| 60,000  24,000  28,000  85,000 | | NVD  CS  CS  CS | 36.5  36  33+3  36 | No  No  Yes  Yes | Gammagard/Gamunex  Gammagard  Omigirm  Gamunex | 1  1  1  1.5 | 17  14  16  16 | 18  22  17  20 | HPA-1a  HPA-1a  HPA-1a  HPA-1a | 1^st^ treated pregnancy  2^nd^ treated pregnancy  3^nd^ treated pregnancy  4^nd^ treated pregnancy |
|  | |  |  |  |  |  |  |  |  | Patient 2 |
| 14000 | |  |  |  |  |  |  |  | HPA-1a  + HPA-3 | 1^st^ affected not treated  pregnancy |
| 347,000 | | NVD | 39+2 | No | Gamunex | 1 | 16 | 23+5 | HPA-1a  + HPA-3 | 1^st^ treated pregnancy |
| 334,000  159,000 | | NVD  NVD | 39+6  39+2 | No  No | Gamunex  unknown | 1  1 | 15  12 | 24  26 | HPA-1a  + HPA-3  HPA-1a  + HPA-3 | 2^nd^ treated pregnancy    3^nd^ treated pregnancy |
|  | |  |  |  |  |  |  |  |  | Patient 3 |
| 8000 | |  |  |  |  |  |  |  | HPA-1a | 1^st^ affected not treated  pregnancy |
| 38,000  100,000  40,000  97,000 | | CS  NVD  NVD  NVD | 38  39  41  38+1 | No  No  No  Yes | Omrigam  Omrigam  Omrigam  Omrigam | 1  1  1  2 | 14  20 | 24  18 | HPA-1a  HPA-1a  HPA-1a  HPA-1a | 1^st^ treated pregnancy  2^nd^ treated pregnancy  3^rd^ treated pregnancy  4^th^ treated pregnancy |
|  | |  |  |  |  |  |  |  |  | Patient 4 |
| 148000 | |  |  |  |  |  |  |  | HPA-5 | 1^st^ affected not treated  pregnancy |
| 87000 | | CS | 38 | No | Unknown | 1 | 11 | 27 | HPA-5 | 1^st^ treated pregnancy |
| 182000  255000 | | CS  CS | 37+4  38+3 | No  No | Unknown  Unknown | 1  1 | 12  13 | 25  25+4 | HPA-5  HPA-5 | 2^nd^ treated pregnancy  3^rd^ treated pregnancy |
|  | |  |  |  |  |  |  |  |  | Patient 5 |
| 225000 | | CS | 37+3 | No | Unknown | 1 | 7 | 30+1 | HPA1a+  HPA-3 | 1^st^ treated pregnancy |
| 289000  289000 | | CS  CS | 36+5  36 | No  No | Unknown  Gammagard | 1  1 | 18  17 | 18  18 | HPA-1a+ HPA-3  HPA-1a+ HPA-3 | 2^nd^ treated pregnancy    3^rd^ treated pregnancy |
|  | |  |  |  |  |  |  |  |  | Patient 6 |
|  | | CS | 38 | No | Gamunex | 1 | 11 | 29 | HPA-3+HPA-5 | 1^st^ treated pregnancy |
| 359000 | | NVD | 38+4 | No | Gamunex | 1 | 9 | 26 | HPA-3+HPA-5 | 2^nd^ treated pregnancy |
| 313000 | | NVD | 38+5 | No | Gamunex | 1 | 12 | 25 | HPA-3+HPA-5 | 3^rd^ treated pregnancy |
| 268000 | | NVD | 39+4 | No | Unknown | 1 | 13 | 30 | HPA-3+HPA-5 | 4^th^ treated pregnancy |
|  | |  |  |  |  |  |  |  |  | Patient 7 |
| 15000 | |  |  |  |  |  |  |  | HPA-1a | 1^st^ affected not treated  pregnancy |
| 191000 | | CS | 38 | No | Unknown | 1 | 11 | 27 | HPA-1a | 1^st^ treated pregnancy |
| 250000  143000  236000 | | CS  NVD  CS  CS | 36  37  31+4  38 | No  No  No  No | Unknown  Unknown  Unknown  Unknown | 1  1  1  1 | 14  15  11  11 | 22  22  20  28 | HPA-1a  HPA-1a  HPA-1a  HPA-1a | 2^nd^ treated pregnancy  3^rd^ treated pregnancy  4^tg^ treated pregnancy  5^th^ treated pregnancy |
| 47,000  60,000  250,000  238,000 | | CS  CS  CS | 36  36  37 | Yes  Yes  Yes | Unknown  Unknown  Gamunex | 2  2  2 | 16  10  9 | 20  16  18 | HPA-3  HPA-3  HPA-3  HPA-3 | Patient 8  1^st^ affected not treated  Pregnancy  1^st^ treated pregnancy  2^nd^ treated pregnancy  3^rd^ treated pregnancy |
| 10,000  110,000  270,000  11,000 | | NVD  CS  CS | 42  39  40 | No  Yes  No | Unknown  Unknown  Unknown | 1  1  1 | 6  16  17 | 36  23  23 | HPA-3  HPA-3  HPA-3  HPA-3 | Patient 9  1^st^ affected not treated  Pregnancy  1^st^ treated pregnancy  2^nd^ treated pregnancy  3^rd^ treated pregnancy |
| 180,000  90,000  87,000  61,000  39,000 | | CS  CS  CS  CS | 34+3  38  38  40 | No  No  No  No | Unknown  Unknown  Unknown  Unknown | 1  1  1  1 | 16  18  16  18 | 18  20  22  22 | HPA-1a  HPA-1a  HPA-1a  HPA-1a  HPA-1a | Patient 10  1^st^ affected not treated  pregnancy  1^st^ treated pregnancy  2^st^ treated pregnancy  3^rd^ treated pregnancy  4^th^ treated pregnancy |
| 50,000  229,000  400,000  250,000 | | NVD  NVD  NVD | 40  40  40 | No  No  No | Unknown  Unknown  Unknown | 1  1  1 | 18  20 | 22  20  20 | HPA-5+HPA-15  HPA-5+HPA-15  HPA-5+HPA-15  HPA-5+HPA-15 | Patient 11  1^st^ affected not treated  pregnancy  1^st^ treated pregnancy  2^nd^ treated pregnancy  3^th^ treated pregnancy |
|  | Patients E and F were treated in their first pregnancies because FNAIT was diagnosed due to family history. GA, gestational age; CS, cesarean section; NVD, normal vaginal delivery; N/A, data not available | | | | | | | | | |
